# Underreporting and Missed Opportunities for Uptake of Intermittent Preventative Treatment of Malaria in Pregnancy (IPTp) in Mali

**DOI:** 10.1371/journal.pone.0160008

**Published:** 2016-08-05

**Authors:** Emily A. Hurley, Steven A. Harvey, Namratha Rao, Niélé Hawa Diarra, Meredith C. Klein, Samba I. Diop, Seydou O. Doumbia

**Affiliations:** 1 Johns Hopkins Bloomberg School of Public Health, Department of International Health, Baltimore, Maryland, United States of America; 2 University of Sciences, Techniques, and Technologies of Bamako, Faculty of Medicine and Odontostomatology, Bamako, Mali; Centers for Disease Control and Prevention, UNITED STATES

## Abstract

**Objectives:**

To identify factors contributing to low uptake of intermittent preventive treatment of malaria in pregnancy with sulfadoxine-pyrimethamine (IPTp-SP) in rural Mali.

**Methods:**

We conducted secondary data analysis on Mali’s 2012–2013 Demographic and Health Survey (DHS) to determine the proportion of women who failed to take IPTp-SP due to ineligibility or non-attendance at antenatal care (ANC). We also identified the proportion who reported taking other or unknown medications to prevent malaria in pregnancy and those who did not know if they took any medication to prevent malaria in pregnancy. We conducted qualitative interviews, focus groups and ANC observations in six rural sites in Mali’s Sikasso and Koulikoro regions to identify reasons for missed opportunities.

**Results:**

Our secondary data analysis found that reported IPTp-SP coverage estimates are misleading due to their dependence on a variable (“source of IPTp”) that is missing 62% of its data points. Among all women who gave birth in the two years prior to the survey, 56.2% reported taking at least one dose of IPTp-SP. Another 5.2% reported taking chloroquine, 1.9% taking another drug to prevent malaria in pregnancy, 4.4% not knowing what drug they took to prevent malaria, and 1.1% not knowing if they took any drug to prevent malaria. The majority of women who did not receive IPTp-SP were women who also did not attend ANC. Our qualitative data revealed that many health centers neither administer IPTp-SP by directly observed therapy, nor give IPTp-SP at one month intervals through the second and third trimesters, nor provide IPTp-SP free of charge. Women generally reported IPTp-SP as available and tolerable, but frequently could not identify its name or purpose, potentially affecting accuracy of responses in household surveys.

**Conclusion:**

We estimate IPTp-SP uptake to be significantly higher than stated in Mali’s 2012–13 DHS report. Increasing ANC attendance should be the first priority for increasing IPTp-SP coverage. Reducing cost and access barriers, ensuring that providers follow up-to-date guidelines, and improving patient counseling on IPTp-SP would also facilitate optimal uptake.

## Introduction

Malaria in pregnancy poses serious risks to the mother, fetus and newborn, including increased risk of maternal anemia, low birth weight and neonatal mortality [1–3]. In 2012, the World Health Organization issued new guidelines for intermittent preventative treatment of malaria in pregnancy (IPTp), recommending that pregnant women be given sulfadoxine-pyrimethamine (SP) at monthly antenatal care (ANC) visits beginning in the second trimester of pregnancy and continuing up until delivery [4]. As a response to these guidelines, the Malian Ministry of Health updated its IPTp recommendations in 2013, calling for a minimum of three doses of IPTp-SP, given monthly from the second trimester up until delivery [5]. These guidelines replace Mali’s previous recommendations for a minimum of two doses of IPTp-SP within a window beginning in the second trimester and ending at the start of the ninth month of pregnancy.

Throughout sub-Saharan Africa, IPTp-SP coverage rates lag behind national targets [6,7]. Sub-optimal ANC attendance is thought to account for some but not all of the coverage gap. According to a recent systematic analysis of 58 household surveys in sub-Saharan Africa, the median proportion of women attending three or more ANC visits was 76.6% [7]. For countries like Mali, which have conducted a Demographic and Health Survey (DHS) seven or more years after adopting an initial IPTp-SP policy, the median proportion of women who took of two or more doses of SP (IPTp-SP2+) was only 29.6%.

DHS data suggests a particularly large coverage gap in Mali, despite a national policy that provides free IPTp-SP at ANC (Table 1) [8,9]. The DHS reports that while 75.0% of women who gave birth in the last two years attended one or more ANC visit, only 19.9% took IPTp-SP2+. The discrepancy is especially pronounced in rural areas, where the vast majority of the population resides. While 71.1% of rural women attended ANC, only 29.6% reported taking a single dose of IPTp-SP (IPTp-SP1), and just 15.3% reported taking IPTp-SP2+.

**Table 1:** Percentage of pregnant women attending antenatal consultations (ANC) and receiving sulfadoxine-pyrimethamine (SP) through ANC in Mali, as presented by the Demographic and Health Survey (2012–2013).

	Total (National)	Rural	Urban
Attended ANC one or more times	75.0	71.1	93.7
Took IPTp-SP1, received at an ANC visit	34.6	29.6	65.0
Attended ANC two or more times	70.1	64.7	90.9
Took IPTp-SP2+, for which at least one dose was received at an ANC visit	19.9	15.3	37.9

Research throughout sub-Saharan Africa has identified health system, household and individual barriers to IPTp-SP uptake. In the health systems realm, it is clear that stock-outs, ANC user fees, disorganized modes of delivery, staff shortages, poor patient counseling, and health worker misconceptions about how and when to administer IPTp-SP can impact uptake [10–17]. On the household level, women’s decision-making and economic power contribute to utilization of ANC services and pharmaceuticals, including IPTp-SP [12]. Individual-level factors related to uptake include knowledge about IPTp-SP, education level, socioeconomic status, timing of ANC visits and number of ANC visits [12,16,18,19].

In Mali specifically, previous studies have revealed the most significant barriers to IPTp-SP at the health system level, including poor patient counseling and inconsistent IPTp-SP administration practices [11,20–22]. Notably, studies conducted by Webster, Hill and colleagues found that IPTp-SP was rarely administered by directly observed therapy as mandated by national policy [20,22]. While these studies shed light on the IPTp-SP situation in Mali, they were all conducted before administration guidelines changed from two doses up until the ninth month of pregnancy to a minimum of three at one month intervals up until delivery. Further, these studies are limited in that they were all conducted in a single district of the Segou region.

To better understand factors contributing to poor IPTp-SP uptake in Mali, we conducted a secondary data analysis on Mali’s 2012–2013 Demographic and Health Survey (DHS) to determine the proportion of women who failed to take IPTp-SP due to ineligibility or non-attendance at ANC. We also conducted a qualitative study in six rural sites in Mali’s Sikasso and Koulikoro regions to describe health systems and sociocultural barriers to uptake.

## Methods

### Secondary data analysis

Using the 2012–2013 Mali DHS maternal health dataset in Stata version 12 [23], we ran cross tabulations at each point where a woman could miss an opportunity to take or report having taken IPTp. First, we filtered out women who never attended ANC and those who attended only during their first trimester (prior to IPTp eligibility). Next, we examined the proportion of women who reported that they (a) took at least one dose of IPTp-SP, (b) took chloroquine as IPTp, (c) took an unknown drug to prevent malaria in pregnancy, (d) took another drug for to prevent malaria in pregnancy, (e) did not know if they took any drug to prevent malaria in pregnancy, and (f) did not take any drug to prevent malaria in pregnancy. Among women who reported having taken IPTp-SP1 and were eligible for a IPTp-SP2 (those who had two or more ANC visits after their first trimester), we identified the proportion who reported that they (a) took the second dose, (b) did not take the second dose, and (c) did not know if they took the second dose.

To be consistent with the DHS report, we restricted analysis to women who gave birth in the two years prior to the survey. For each analysis, we examined the national sample, and the subset of women from rural areas. All statistics were weighted using DHS methodology [24].

### Qualitative study

We conducted 28 in-depth interviews (IDIs), 26 focus-group discussions (FGDs) and 29 ANC observations across six rural health zones across Sikasso and Koulikoro. These two regions offer advantages in the study design as they are geographically different from where similar studies have been conducted before (Segou) [11,12], yet have similar IPTp uptake estimates to the national averages [8]. We began by purposively selecting two rural health zones within each region, one served by a larger health center accessible by paved road in a central town or population center, and another served by a smaller health center deep in a rural village and far from a population center or paved road. Within these sites, we purposively sampled community members who could offer a range of perspectives on IPTp, including pregnant women, family members, health workers and community leaders (Table 2).

**Table 2:** Qualitative data collection by region, method, and type of participant.

	Koulikoro Region	Sikasso Region	Total
**In-depth Interviews (IDIs)**	**15**	**22**	**37**
Pregnant or breastfeeding woman	6	9	**15**
*Matrone* or auxiliary midwife	2	2	**4**
Pharmacist	2	1	**3**
Physician / Health center Chief	-	4	**4**
Health management committee (ASACO) president	1	-	**1**
Community health volunteer	1	1	**2**
NGO worker	-	1	**1**
Mayor	-	1	**1**
District Health Official	3	3	**6**
Focus Group Discussions (FGDs)*	**12**	**14**	**26**
Elderly women	3	5	**8**
Teachers	1	2	**3**
Community health volunteers	2	3	**5**
Husbands	3	2	**5**
Village officials	1	1	**2**
Pregnant or breastfeeding women	2	-	**2**
Health management committee (ASACO)	-	1	**1**
**Observations**			
ANC visits	14	15	**29**

* Numbers presented refer to number of focus groups, not number of participants.

We determined we had reached data saturation in IDIs and FGDs with community members in the original four sites, as iterative analysis revealed recurring, developed themes both within and between sites [25,26]. However, because procedures for administering IPTp-SP differed significantly in each of the first four health facilities we observed, we decided to observe additional sites to better characterize that variability. Thus, we added one additional observation site per region. We also determined that themes regarding health system factors warranted triangulation from higher-level health officials. Thus, we added six key informant interviews with leaders at district- and regional-level reference hospitals toward the end of the study.

The data collection team included four Malian women trained in qualitative research methods (one sociologist and three physicians) and the first author, an American doctoral student fluent in Bambara. IDIs and FGDs with community members were semi-structured and included topics on a) health, illness prevention and use of medications during pregnancy, b) experiences with ANC and IPTp-SP, and c) perceived barriers to IPTp-SP uptake. The original English versions of the IDI and FGD guides are included as an appendix (S1 Appendix). During key informant interviews, district and regional level health officials were asked about content and implementation of IPTp-SP policy and their views on factors contributing to uptake. The original English versions of the IDI and FGD guides are included as an appendix. IDIs and FGDs were conducted in Bambara, the local language, with the exception of some key informants and clinicians who preferred French. Participants were interviewed in private locations within the community (typically, the participant’s home or place of work). All IDIs and FGDs were audio-recorded, then translated and transcribed into French.

The ANC observations were conducted in order to characterize the context and processes of IPTp-SP administration at each health center. Based on pre-testing, we determined that with two data collectors and a purposive sample five patients per center, we could produce a thorough and internally consistent description of that health center’s processes. The purposive sampling was intended to capture women at different points in their pregnancy. Data collectors observed each participant for the entirety of her ANC visit and completed a structured observation form covering the patient-provider interaction, clinical exam, content of patient counseling, and acquisition/administration of pharmaceuticals. Researchers also took unstructured notes detailing the events and context of the visit. Additionally, we reviewed maternity and pharmacy record books at each health center for SP stock and distribution information.

Analysis of textual and observational data was an iterative, collaborative process. Interviewers wrote reflective memos after each data collection session and discussed developing themes at regular debriefing meetings [27]. Throughout data collection, we added and adjusted questions on IDI and FGD guides based on previous IDIs, FGDs, and debriefings. Transcripts and observation notes were uploaded into ATLAS.ti version 7 for descriptive coding [28]. The research team began coding inductively and periodically revised the codebook as new themes emerged. A sample of transcripts were double-coded to ensure consistency between researchers. Multiple members of the research team reviewed and discussed data outputs of each code to summarize findings.

### Ethical considerations

The Institutional Review Boards of the Johns Hopkins Bloomberg School of Public Health and the Faculty of Medicine, Pharmacy, and Odontostomatology at the University of Sciences, Techniques, and Technologies of Bamako approved the study. Informed consent was obtained from all participants of IDIs, FGDs and observations. Interviewers signed consent forms to document oral consent, which was collected in order to overcome literacy limitations and ensure participant anonymity.

## Results

Together, our secondary data analysis and qualitative study reveal an underestimate of IPTp uptake in Mali’s DHS report, the impact of ANC attendance in IPTp uptake, questionable validity of reporting IPTp through household surveys, and challenges related to the administration of IPTp through ANC.

### Secondary data analysis

#### Underestimate of IPTp uptake in DHS report

**Fig 1:**
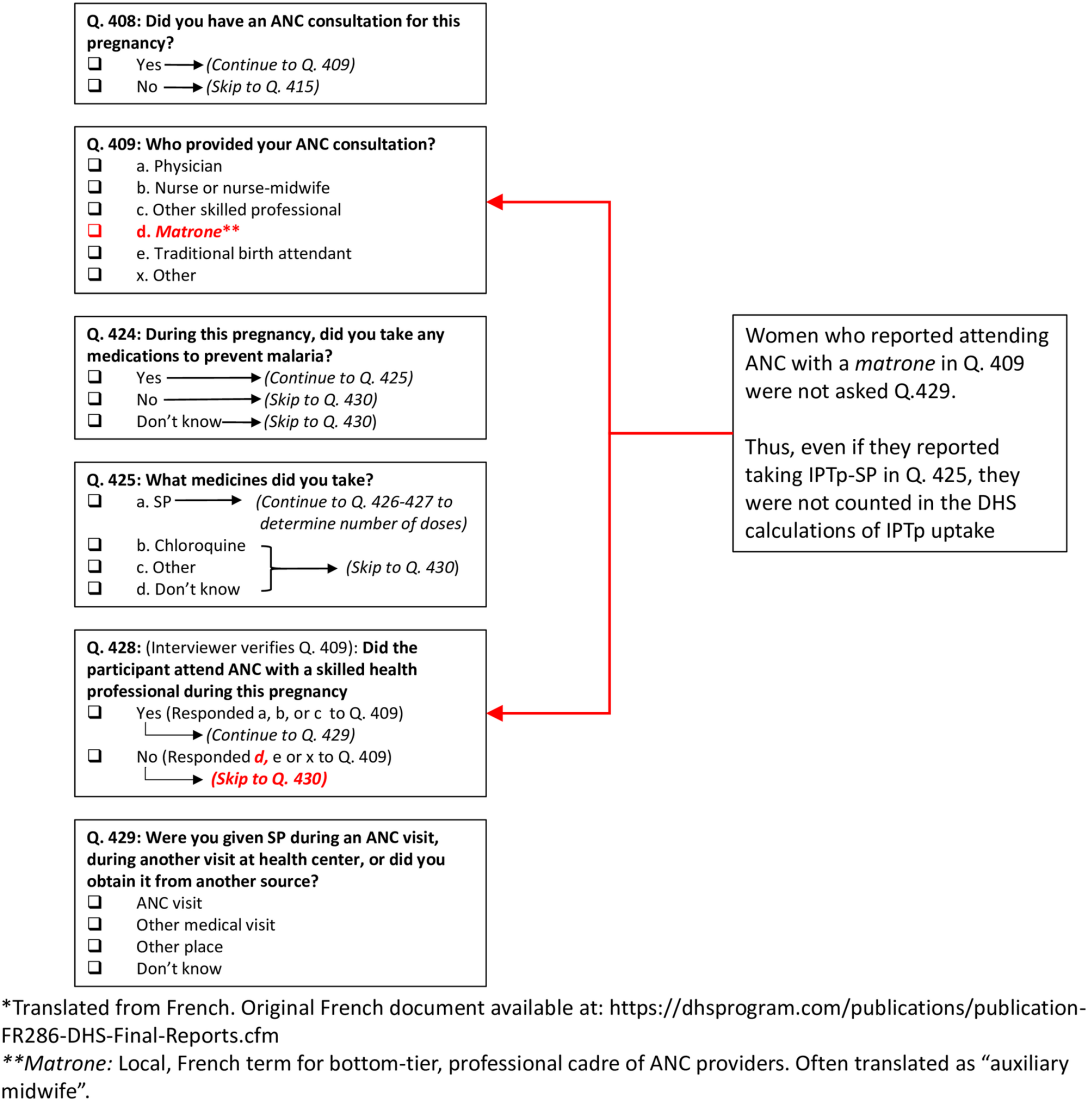
Excerpts of Mali’s 2012–2013 DHS questionnaire, illustrating how a skip pattern resulted in an underestimation of IPTp-SP.

**Fig 2:**
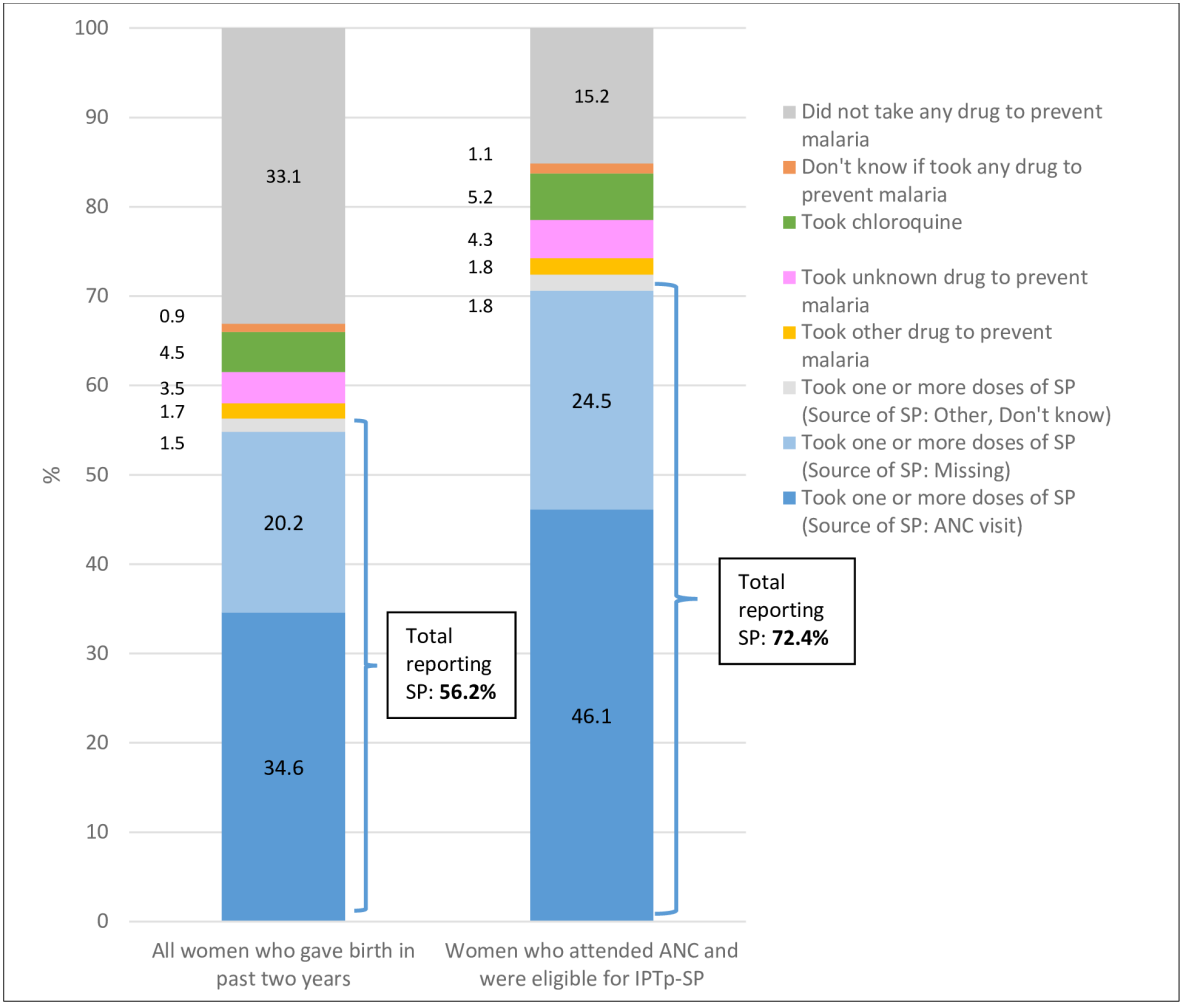
Distribution of uptake (IPTp-SP1+) reported by women who gave birth in the past two years in Mali’s 2012–2013 DHS (“source of IPTp-SP” variable excluded).

**Fig 3:**
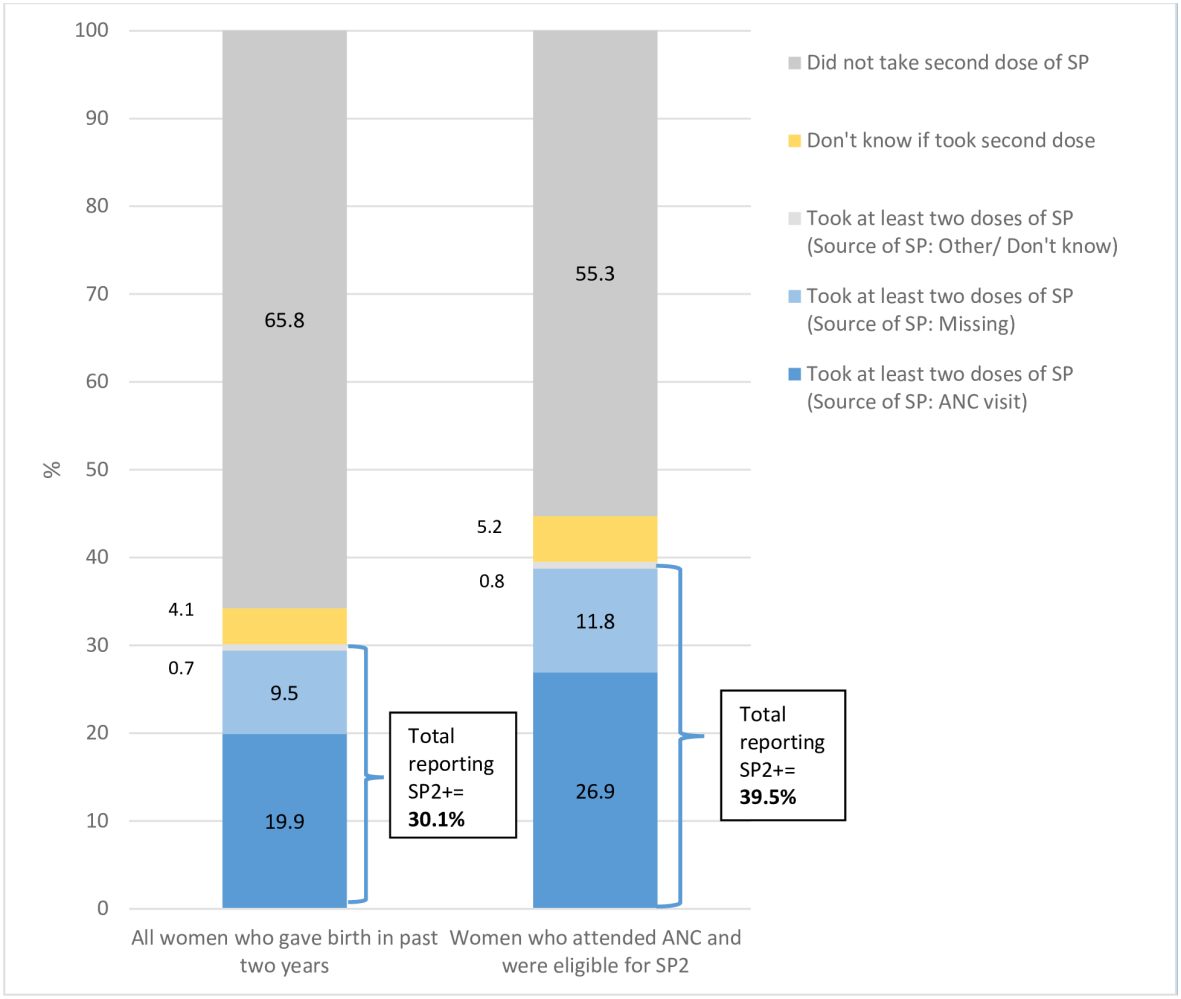
Distribution of uptake of (IPTp-SP2+) reported by women who gave birth in past two years in Mali’s 2012–2013 DHS (“source of IPTp-SP” variable excluded).

Our analysis suggests that IPTp uptake in Mali is likely higher than the most recent DHS report suggests. Fig 1 illustrates how an erroneous screening item in the survey ultimately produced these underestimates. On DHS survey question 409, women who reported attending ANC are asked the type of health provider they saw. Answer choices include a) physician or nurse, b) nurse-midwife, c) other skilled professional, d) *matrone* (typically translated as “auxiliary midwife”), e) traditional birth attendant and x) other. Women are also asked whether they took any medication to prevent malaria in pregnancy (question 424) and, if so, whether that medication was SP, chloroquine, or something else (question 425). Based on each respondent’s answer to question 409, a screening item (question 428) then divides respondents into those seen by a “skilled provider” (physician, nurse, nurse-midwife, or other skilled professional) or a “non-skilled provider” (*matrone*, traditional birth attendant, other). Only women classified as having received ANC from a “skilled provider” according to this screening item (question 428) are then asked question 429: *where* they received IPTp (answer choices: ANC, other medical visit, other). This variable, which we will refer to as “source of IPTp-SP,” is the basis of the IPTp-SP estimates that appear in the DHS report (“women who received IPTp *during the course of an ANC visit*”). Over 35% of women who attended ANC reported a *matrone* as their ANC provider. As a consequence of the screening item, most of these women have missing data for the “source of IPTp-SP” variable. Thus, these respondents were left out of the final DHS calculations for IPTp-SP coverage *even if* they had earlier reported (in question 425) that they received IPTp-SP. The classification of *matrones* as “non-skilled providers” is misleading, as 33.9% of pregnant women who reported taking IPTp-SP also reported attending ANC with a *matrone*. When we omitted the “source of IPTp-SP” variable from the calculations, we found that a total 56.2% women who gave birth in the last two years reported having taken IPTp-SP, a figure 62% higher than the DHS estimate of 34.6% (Fig 2). We also found that 29.9% of women received their second dose of SP IPTp, whereas the DHS report estimates 19.9% (Fig 3).

**Fig 4:**
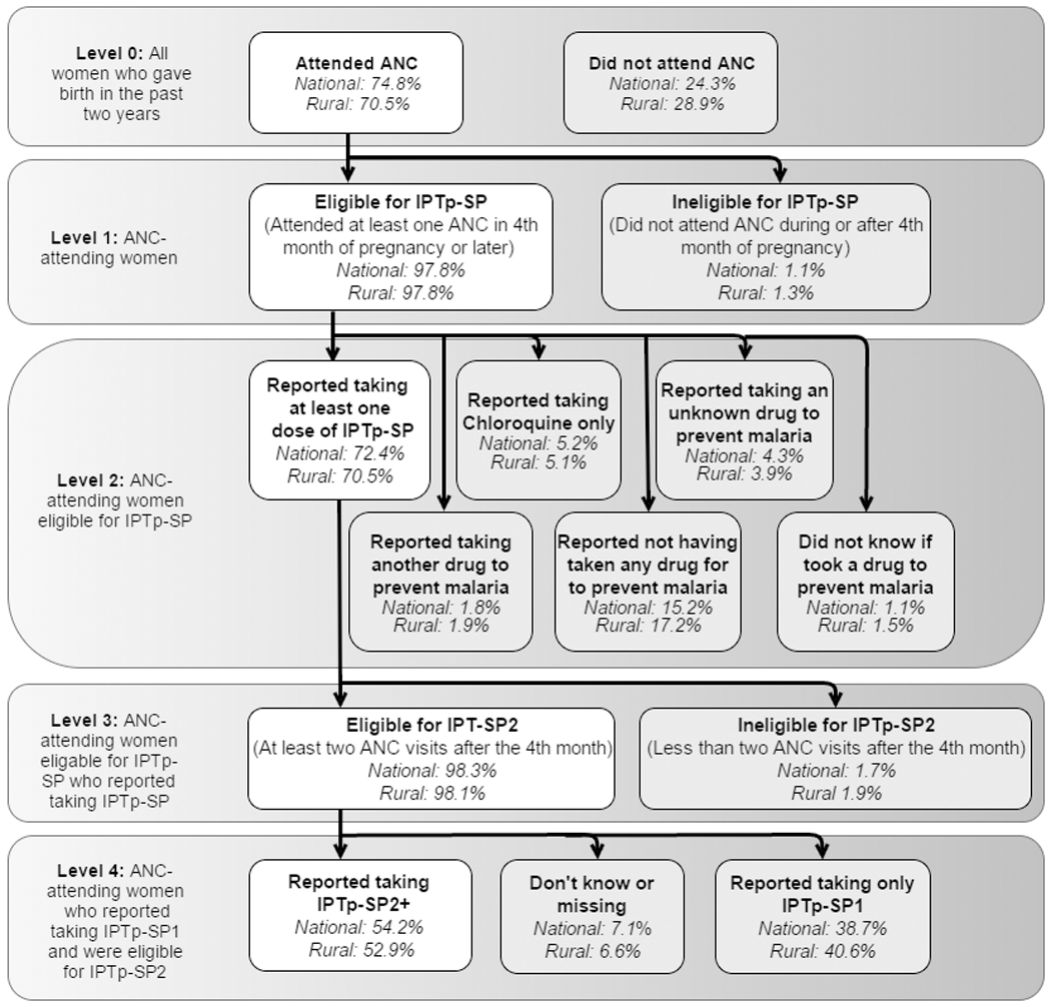
Breakdown of DHS statistics on missed opportunities for IPTp-SP uptake among women having a live birth in the previous two years in Mali, nationally and in rural areas.

While 72.4% of eligible women who attended ANC reported taking at least one dose of IPTp-SP, only 15.2% reported *not* taking any medication to prevent malaria in pregnancy (Figs 2 and 4). The remaining reported taking an unknown drug to prevent malaria (4.3%), another drug to prevent malaria (1.8%), taking chloroquine (5.2%), or not knowing if they took IPTp (1.1%).

#### Impact of ANC attendance

Using the DHS ANC and IPTp items without the screening variable, we estimated the percentage of women who fail to receive IPTp-SP because of non-attendance at ANC or IPTp ineligibility (Fig 4). Nationally, 24.3% of women (28.9% in rural areas) did not make even a single ANC visit. Women who did not attend ANC made up the majority (51.8% overall; 56.1% in rural areas) of those who reported not receiving IPTp-SP. The vast majority of women who attended ANC had at least one visit after their first trimester (97.8% overall, 97.8% in rural areas), making them eligible for IPTp-SP. For eligible women who did attend ANC, 72.4% nationally (70.5% in rural areas) reported taking at least one dose of IPTp-SP.

#### Remaining gaps

Despite DHS underestimates of IPTp-SP uptake, it is evident that real gaps remain in IPTp-SP delivery at the health system level. Our analysis shows that 15.2% of eligible, ANC-attending women claimed to have not taken any drug to prevent malaria in pregnancy (Fig 4). Further, over 98% of women who reported taking their first dose of IPTp-SP were eligible for a second dose, meaning they attended at least two ANC visits after their first trimester. Yet among them, just 54.2% (52.9% in rural areas) reported receiving their second dose, 38.7% (40.6% in rural area) reported not receiving their second dose and 7.1% (6.6% in rural areas) did not know or had missing data.

### Qualitative study

Data from IDIs, FGDs and ANC observations revealed factors influencing IPTp-SP uptake including barriers to attending ANC, inconsistent implementation of national IPTp-SP administration guidelines and poor patient counseling. Further, we found that during discussions of women’s health, many women had difficulty naming or describing SP.

#### Barriers to attending ANC

Health workers considered non-attendance or late attendance at ANC among the top barriers to IPTp-SP uptake. While women and family members saw ANC as valuable, they noted cost and distance as significant barriers to care-seeking. With limited or no control over household finances, most women were dependent on husbands or other male family members, who themselves often found it difficult to pay for ANC user fees and prescriptions.

“We are farmers—some have the means, others do not—and their wives cannot go to ANC. We have these difficulties.

*(Husband*, *FGD*, *Koulikoro site #1)*.

Reluctance to reveal pregnancy early on may also inhibit ANC care-seeking. According to participants, there is shame (*‘maloya’* in Bambara) associated with others—even family members—learning that one is pregnant. While women themselves did not mention *maloya*, community health workers, husbands and village elders reported that it can cause women to delay ANC. During a FGD, one husband claimed that shame was a “big problem” in regards to ANC care-seeking, while another explained:

“When you go to the ANC, you go in a group. You will be seen, and that is why the woman are shamed”

*(Husband*, *FGD*, *Koulikoro site #1)*

#### Inconsistent implementation of national guidelines

Women who do attend ANC may also miss the opportunity to benefit from IPTp-SP due to inconsistent implementation of national administration guidelines. Our observational data revealed that SP was administered by directly observed therapy at only three out of our six sites. Only one of these sites practiced directly observed therapy consistently across all eligible participants observed (Table 3). Citing the misperception that the SP should not be taken on an empty stomach, health workers at the other three sites instructed women to take it at home after a meal, opening up the possibility that they could later forget or decide not to take the pills. Among women in IDIs who recalled having been offered SP, all reported having taken it and finding it tolerable. Some however, said that ‘‘other women” who “don’t like medicines” might throw the pills out when given them to take home.

**Table 3:** IPTp administration practices at six rural health centers in the Koulikoro and Sikasso regions of Mali.

	Administered SP by directly observed therapy	Considered women eligible for IPTp through the 9th month	Explained the purpose of IPTp to patients
	Data Source: ANC Observations	Data Source: Health worker interviews	Data Source: ANC Observations
Koulikoro Site 1	No	No	Yes, in a group education session
Koulikoro Site 2	Typically, but allowed one woman who said she hadn’t eaten to take it home	No	No
Koulikoro Site 3	No	Unclear	No
Sikasso Site 1	Yes, but did not consistently observe the women taking it at the health center	No	For 2/5 participants
Sikasso Site 2	No	No	For 2/5 participants
Sikasso Site 3	Yes	Yes	No

Though the most recent national guidelines specify that IPTp-SP may be given up until delivery [5], health workers at four of the six sites withheld SP from women in their 9^th^ month of pregnancy, reporting that it would result in birth defects or other problems (Table 3).

“At first, there is no heartbeat and the infant has not started moving. Later, it’s more than an embryo and medications during this stage can cause deformities. We do not prescribe SP at the 9^th^ month since it causes heart problems.”

*(Nurse-midwife*, *IDI*, *Koulikoro site #1)*

Further, while national policy states that IPTp should be given free, cost or perceived cost may have a negative impact on uptake. Our data revealed that sometimes women are charged, or have the impression of being charged for IPTp-SP. At one site for example, women were given the first dose free but required to pay for subsequent doses. At two others, SP was added to a long costly prescription list, and health workers did not consistently specify that SP could be obtained for free. In an IDI, one provider explained that women would sometimes bypass the pharmacy altogether—including the free SP—if the lump sum on their prescription list became too expensive. In cases of stock-out, health workers reported women would have to buy SP at a private pharmacy, often adding to the burden of cost and distance for ANC services. However, according to health worker IDIs, stock-outs are a rare occurrence. Our review of pharmacy records did not reveal significant periods of stock-outs, though many of these records were incomplete or unavailable. However, many key informants at the regional-level hospitals did mention stock-outs as a significant barrier to effective IPTp-SP. One considered stock-outs as “the main weakness” of their regional IPTp-SP program.

#### Poor IPTp-related patient counseling

Poor patient counseling about IPTp-SP may also impact uptake, especially when directly observed therapy is not consistently practiced. While SP was offered to all women considered eligible during our ANC observations, patient-provider interactions about IPTp were usually brief. Women were often instructed to take the pills with little or no explanation about their purpose:

The midwife asks the patient, “Did you eat breakfast? Did you eat enough?” The patient says she had had enough to eat that morning. The midwife gives her three tablets of SP and tells the patient to take them. The patient chews the pills and washes them down with the water offered to her. It all happened very quickly and nothing was explained to the patient about what the pills were for.

*(Observer field notes*, *Sikasso site #2)*

Health workers in only three of our observation sites explained the purpose of SP to patients, yet in two of these, the explanation was only offered to two of the five patients we followed (Table 3). In one site where women were given SP to take home, they were told it was “very important” but not why. In another, the midwife did not explain the purpose of SP, but yelled at a departing patient “Make sure you take it, because some women don’t!” In IDIs and FGDs as well, women gave mixed reports about the extent to which providers explained IPTp-SP. While one patient claimed her providers “highly insisted on SP” during her ANC visit, another claimed, “I received these treatments, but they did not tell me why.”

#### Low emphasis on IPTp in discussions of women’s health

Poor patient counseling may have also contributed to the low emphasis women and family members placed on IPTp-SP in IDIs and FGDs. Women considered malaria among the biggest threats to health during pregnancy. In discussions of malaria prevention, they spoke extensively about the importance of mosquito nets, yet rarely mentioned IPTp-SP. When probed by the interviewer, a number of women were able to recall “the three white pills” given to them at ANC, but often could not explain their purpose.

During discussions of pharmaceuticals used during pregnancy, women typically spoke about iron to a greater extent than SP. Many participants who could not explain the use of SP could do so for iron. The local name for iron, “*joli caya fura*” or “the medicine that increases blood,” clearly signifies its commonly understood purpose, possibly enhancing its recall among participants. Further, the pill’s distinctive red color is also easily associated with blood. In comparison, SP does not have a Bambara term or a notable color. Most referred to SP as “*furakise den saaba*” (the three pills), “*furakise jeman*” (the white pills) or less frequently, “*sumaya fura*” (malaria medicine). In one Sikasso site, women commonly referred to the drug given for IPTp as “CTA,” the French acronym for artemisinin-based combination therapy, even though women in our ANC observations at that site were given SP.

## Discussion

Through a secondary data analysis and a qualitative study, we identified three potential points of missed opportunities for IPTp-SP uptake in Mali: (1) The woman does not attend ANC; (2) the woman attends ANC but does not take IPTp-SP; and (3) the woman takes one dose of IPTp-SP but not subsequent doses (Table 4). Additionally, a woman could take IPTp-SP but not recognize that she had taken it or mistakenly report it as a different drug during a later household survey, which may affect the accuracy of DHS statistics on IPTp uptake. Our secondary data analysis suggests that among these, non-attendance at ANC is the biggest contributor to the gap in IPTp-SP uptake.

**Table 4:** Missed opportunities for IPTp-SP uptake or reporting of IPTp-SP uptake among women in rural Mali.

	Magnitude of problem	Contributing factors
	(Secondary data analysis results)	(Qualitative study results)
**Missed opportunities to provide/receive IPTp-SP**		
1. The woman did not attend ANC	24.3% of women did not attend ANC (28.9% in rural areas) 51.8% of those who did not take IPTp-SP did not attend ANC	Access barriers like cost and distance Cultural expectation that a woman should have *maloya*, (“shame”) and not publicly reveal her pregnancy
2. The woman attends ANC but does not take IPTp	15.2% of those who attended ANC and were eligible to receive IPTp-SP reported not taking any medication to prevent malaria (17.4% in rural areas) A small proportion (~1%) were ineligible for IPTp, because they did not attend ANC after their first trimester of pregnancy	Inconsistent directly observed therapy Withholding IPTp-SP from women in their 9^th^ month of pregnancy Cost or perceived cost of SP for patients
3. The woman takes the first dose of IPTp but not a second dose	38.7% of women who took one dose of IPTp-SP and were eligible reported not taking a second dose (40.6% in rural areas)	Starting ANC later in pregnancy due to access barrier or shame, which made some women ineligible, or erroneously considered ineligible for a second dose. The incorrect belief among health workers that IPTp-SP should not be administered in the 9th month of pregnanc Charging for subsequent doses of IPTp-SP
**Missed opportunities to report IPTp-SP uptake**		
The woman receives IPTp but misreports it	Of those women attending ANC and eligible for IPTp-SP, 5.2% reported taking chloroquine as IPTp, 1.8% taking another drug to prevent malaria, 4.3% did not know what drug they took to prevent malaria, and 1.1% did not knowing if they took a drug to prevent malaria.	Poor patient IPTp-related counseling The lack of a name for SP in the local language

In examining the DHS dataset, we found that the IPTp-SP uptake figures presented in the Mali’s DHS report are misleading. These figures incorporate a screening item (“source of IPTp”) that was intended to ensure that when a respondent claimed to have taken IPTp-SP, she was referring to IPTp-SP administered by a qualified ANC provider. While the use of this screening item is well-intentioned, it means that IPTp-SP uptake statistics presented in Mali’s report are probably considerably underestimated. The root of the problem was the failure to classify *matrones* as “skilled providers” in the screening item, despite the fact that approximately 34% of all pregnant women who claimed to have taken IPTp-SP attended an ANC conducted by a *matrone*. Calculating uptake without the “source of IPTp-SP” variable places estimates closer to those from DHS reports in neighboring countries such as Niger and Sierra Leone as well as to estimates reported by Hill and colleagues in Mali’s Segou Region [20,29,30]. Of Hill and colleagues’ total sample of pregnant women, 47.8% received IPTp-SP1 and 28.6% received IPTp-SP2+ [20].

In many rural villages, *matrones* are the only skilled provider that women can access. Though their role as a “skilled provider” was overlooked in IPTp-SP calculations, *matrones* are classified as skilled providers in an earlier section of the DHS report (“Prenatal Care”, section 8.1.1, p.105). Further, Mali’s most recent comprehensive policy document for reproductive health, published in 2004, indicates that *matrones* are fully qualified not only to conduct ANC, including preventative services, but also to assist at deliveries [31]. Yet in a table from that document indicating “activities and tasks” of different cadres of providers, IPTp-SP is indicated as an activity of doctors, nurses, and nurse-midwives, but not *matrones*. It is possible that this document may have influenced misclassification of *matrones* in the DHS IPTp-SP calculations. To our knowledge, the Ministry of Health has yet to publish an updated policy specifically indicating that *matrones* are fully qualified to administer IPTp-SP as part of the standard package of ANC services they provide. Nevertheless, if *matrones* are considered qualified to assist women during labor and delivery, it seems reasonable to assume that they are also qualified to administer IPTp.

According to our secondary data analysis, non-attendance at ANC is the biggest contributor to the gap in IPTp coverage. Consistent with our qualitative data, an abundance of literature has identified cost and distance as major barriers to ANC care-seeking [32–34]. In Mali, other researchers have elaborated on the sociocultural concept of *maloya* (“shame”) as it relates to ANC care-seeking in rural areas. A woman who reveals her pregnancy, according to anthropologist Margaret Holten, announces her sexual behavior and makes herself and her unborn child vulnerable to curses from jealous or evil neighbors [35]. The *maloya* related to revealing pregnancy may deter women from attending ANC. Moreover, ANC care itself can be considered inappropriate or shameful, specifically if an older woman must agree to be examined by a younger female care provider [35,36].

Yet even if a woman makes it to ANC, she may still miss the opportunity to benefit from IPTp, particularly the second dose. Over 55% of women who attended ANC reported not taking IPTp-SP2, including nearly 39% of women who took IPTp-SP1. Similar to earlier studies, we identified health systems barriers such as failure to administer IPTp-SP by directly observed therapy and charging for IPTp-SP [20,22]. We also found that many health workers withheld IPTp-SP from women in their ninth month of pregnancy, a practice based upon outdated guidelines [37]. There is evidence however, that providers had misinterpreted recommendations even before the change in guidelines, as Webster and colleagues report providers withholding IPTp-SP from women in their eighth month or last trimester of pregnancy [11]. The failure to administer IPTp-SP to women in later stages of pregnancy may help explain the substantial drop in uptake from the first dose to the second. Unfortunately, the DHS only reports timing of the first ANC visit, making it impossible to estimate IPTp-SP uptake among women who made their second ANC visit during the later stages of their pregnancy. While efforts to increase ANC attendance are certainly needed, improving health worker compliance with national IPTp-SP guidelines is likewise essential to increasing coverage.

It is also possible that some women may have received IPTp-SP and did not accurately report it in the DHS household survey, as 12.4% of those eligible for IPTp-SP reported taking an unknown drug, taking an “other” drug, taking chloroquine, or not knowing if they took any drug to prevent malaria in pregnancy. While we cannot confirm this theory with existing DHS data, our qualitative results suggest that self-reporting of IPTp-SP may be subject to recall bias, as many of our participants could not name or describe SP. Poor knowledge and/or recall may stem from poor patient counseling about IPTp-SP as well as the fact that SP does not have an illustrative name in the local language. In their study on local conceptualizations of pharmaceuticals, Patterson and colleagues also noted the lack of a distinct Bambara name for SP, which participants often confused with other white pills and other pills for curative malaria treatment [38]. Interestingly, we found that 5.2% of women in the DHS who were eligible for IPTp claimed to have taken chloroquine, which was commonly given to pregnant woman as chemoprophylaxis prior to the 2004 WHO instigation of IPTp [39,40]. Mali’s IPTp policy during the time of the DHS survey was to provide SP, thus, it is possible that some respondents who did receive SP mistook it for chloroquine. Media and policy coordination to promote a Bambara name for SP may help improve recall in future surveys, as may showing visual aids or sample medications to survey respondents.

Our results have a number of limitations. Though our focus on rural areas in the two most populous regions in Mali is a strength, we visited a relatively small number of sites. Our findings may not reflect the situation in other areas of the country, in particular the culturally-distinct northern regions that have faced political conflict and population displacement in recent years. Another important consideration is that the study focused on community-level factors, and further research may be needed to uncover more distal factors (on the national and regional level) that may influence IPTp-SP uptake.

Our findings have implications for future DHS surveys as well as malaria prevention programs. The misclassification of “skilled providers” reveals the need for close periodic revision of large surveys, as the nature of what is being measured may change over time. After our research team brought this misclassification to the attention of DHS analysts, the DHS program uploaded to their website revised updates for affected surveys in Burkina Faso (2010), Guinea (2005), Guinea (2012), Mali (2006), and Mali (2012–13) [41].

The corrected DHS calculations are slightly lower than ours, as we present all women who reported IPTp-SP while the DHS still restricts estimates to those who also report attending ANC with a skilled provider (a category that DHS has now corrected to include *matrones*). Though the DHS has made this correction, it is unknown if the current method of restricting data to “skilled providers” effectively captures local variations in the types of providers qualified to administer IPTp-SP across the diverse health system structures of sub-Saharan Africa. For example, the current method of calculating IPTp-SP excludes the possibility that IPTp-SP could be effectively administered by a community health worker or outside of ANC through community-based programs. As pilot interventions of community-based IPTp administration in Nigeria and Uganda have yielded promising results, future scale-up of such programs are a strong possibility [42,43].

IPTp-SP programs are at a critical juncture, as growing concerns about SP resistance make it imperative that the recent increase in recommended doses is widely implemented [4,44]. In Mali, interventions should prioritize increasing ANC attendance or reaching non-attending pregnant women through community-based IPTp-SP administration. Mali’s Ministry of Health and its partners must work to ensure that national guidelines have been thoroughly communicated and are being consistently implemented by health workers at all levels of the health care system. Stakeholders should also explore ways of making SP easier for the population to identify and recall and should strategize with DHS implementers to maximize validity of reporting.

## Supporting Information

S1 Appendix IPTp Mali Interview Guides. Original Interview and Focus Group Guides.
(DOCX)
