# Supplementary material for: Underreporting and Missed Opportunities for Uptake of Intermittent Preventative Treatment of Malaria in Pregnancy (IPTp) in Mali
Source: PLoS One. 2016 Aug 5;11(8):e0160008. doi: 10.1371/journal.pone.0160008 (PMC4975448; doi:10.1371/journal.pone.0160008)
Supplement: S1 Appendix — Original Interview and Focus Group Guides. (DOCX) [file pone.0160008.s001.docx]

**Appendix:**

Underreporting and missed opportunities for uptake of intermittent preventative treatment of malaria in pregnancy (IPTp) in Mali

**Original Interview and Focus Group Guides**

**Instrument #1: Interview/focus group guide for pregnant or recently pregnant women** (page 2)

**Instrument #2: Interview/focus group guide for husbands, mother-in-laws, family members** (page 6)

**Instrument #3: Interview/focus group guide for village leaders** (page 9)

**Instrument #4: Interview guide for village-level health workers** (page 12)

**Instrument #5: Interview guide for Regional and District level health officials** (page 13)

**Instrument #1: Interview/focus group guide for pregnant or recently pregnant women**

*Note to interviewer: Explain to your informant that you are interested in her experience with pregnancy. Explain why she was chosen to be interviewed and that her perspective is very important to you. Emphasize that you want to learn from her, that there are no right or wrong answers, and that she can interrupt you at any time if she has something to say. Let the informant talk about what she thinks is important. Remind her that you will not record her name and that everything you talk about will be kept confidential.*

1. I’d like to start by asking about your current / most recent pregnancy. Please tell me about your pregnancy from the time you realized you were pregnant until now. Take me through a day in your life, from when you wake up in the morning to when you go to bed at night, what exactly do you do? Tell me about what you do/did when you first get up, what you do in the morning, what you do/did around mid-day, what you do/did in the afternoon, what you do/did in the evening, and what you do/did just before you go to bed. (Probe as appropriate based on responses.)

*Note: the purpose of this question is not to develop a detailed ethnography of every aspect of pregnancy, but to see what, if any, aspects of pregnancy related to malaria and prevention the respondent might mention spontaneously. While we are interested in non-malarial and non-biomedical illness that might affect whether a woman receives IPTp1 and IPTp2+, we are not attempting to develop an exhaustive catalogue of everything that can go wrong in pregnancy or that might affect pregnancy outcomes.*

Probes

- How is this different from what you would do before you became pregnant? What things are different? What things are still the same?
- How is the work you do different or the same?
- How is what you do around the house different or the same?
- How are you feeling?

1. Now I’d like to ask you about health during pregnancy. What are the important things to do to stay healthy when you are pregnant?

*Note: We should look for terms in Bambara that encompass “health” in the sense of overall well-being – not just being bio-medically “sick” or “healthy,” but extending to misfortune – things that might be caused by bad luck or jealousy or supernatural forces. Again, the purpose of this is not to develop an exhaustive list of everything that can go wrong in pregnancy, but to understand what might influence decisions about IPTp.*

Probes:

- What do you do to protect your health when you are pregnant?
- What do you do to make sure your baby is born healthy?
- Is there any kind of special care a woman needs when she is pregnant?
- Where do you get that care? (If appropriate, ask about different kinds of care for different things to try to determine if there are certain health issues the informant identifies as appropriate domains for biomedical care versus others that are appropriate domains for other kinds of care.)
- In addition to you yourself, who helps decide about your care during pregnancy?
- What happens if that person/those people are away or cannot be reached?

1. What illnesses can happen during pregnancy?

Probes:

- What illnesses are especially dangerous for the mother or the baby?
- What makes them especially dangerous?
- What can/should a pregnant woman do to keep from getting [name of illness]?
- What did you do / what are you doing to keep from getting [name of illness]?
- If you / a pregnant woman got [name of illness] what should she do?

1. Some people say it’s good to take certain medicines during pregnancy. Some people say pregnant women shouldn’t take medicine. What do you think?

Probes:

*If the interviewee mentions a particular medicine, probe as appropriate: What does this medicine do? What is the purpose of taking it? How often do / did you take it? How often should it be taken?*

- What medicines have you taken during this pregnancy?
- What medicines have you taken in previous pregnancies?

*Again, probe as appropriate: Did it work the way you expected? What happened that you expected? What happened that you did not expect? (We want to get at concerns about or experience with adverse effects, preferably without having to prompt directly)*

- What medicines do you avoid when you are pregnant?
- Why is it important to avoid these medicines?

*Additional probes if they haven’t already come up:*

- What are the occasions/reasons when you would take / have taken medicine during your pregnancy? Do you take medicine only when you are sick?
- Do you ever take medicine to keep from getting sick? If so, when would you take medicine to keep from getting sick?
- How do you decide whether to use a certain medicine when you are pregnant?
  - Do you decide by yourself or do other people help decide?
  - If other people help, who else is involved in the decision?
  - What if that person is not available when you are deciding what to do?

1. Now I’d like to ask you about the care for pregnant women provided by the Health Center (CSCom). What has been your experience with the CSCom during pregnancy?

Probes:

- Have your experiences with the CSCom been good or fair or not good? What has made them good/fair/not good?
- If your sister or a friend were pregnant and asked you if she should go to the CSCom, what would you tell her? Why would you tell her that?
- Besides the CSCom, where else could a pregnant woman go for care during pregnancy? [Note: we need to find the right translation for the word “care” here: if the French word “soin” refers only to the type of care provided by a biomedical establishment, we need to find a word with a broader meaning that would include non-biomedical types of care].
- (As a follow-up to the previous probe): You mentioned X, Y, and Z as places a woman might go for care during pregnancy. How would a woman decide whether / when to go to X, when to go to Y, and when to go to Z?

1. You may know that the CSCom provides a special type of care for pregnant women called antenatal care (ANC). Please tell me about what experience you’ve had with ANC at the CSCom.

Probes:

- What happens at the CSCom [name] where you go for ANC? What do they do during ANC? What do they say?
- What medicines do they give you during ANC visits? What medicines do they want you to take? How do you decide whether to take a medicine they want you to take? (Probe on this point thoroughly: what factors contribute to the informant’s decision to take or not take medicine prescribed by the CS?)
- When the CSCom tells you to take medicine, do they give the medicine they want you to take? Is it free or do you have to pay? If you have to pay, how much does it cost? Is that an affordable price? (If not, what would be an affordable price?)
- When the CSCom tells you to take medicine, do they have the medicine available? (always/sometimes/never) If they do not have it, where would you get it?
- Besides taking medicine, what else did the CSCom tell you to do to keep yourself and your baby healthy during pregnancy? [Note: This question is meant to see whether the woman mentions malaria prevention or ITNs spontaneously. Do not prompt at this point.]
- Besides medicine, did the CSCom give you anything else during your ANC visits? If so, what did they give you? Was there anything you expected them to give you that they did not give you? [Note: This question is also meant to see whether the woman mentions ITNs spontaneously. Ask the next probe only if she does *not* mention ITNs.] Probe: Did the CSCom give you a mosquito net during any of your ANC visits?
- At what point in your current/last pregnancy did you make your first ANC visit? (Ask to verify with ANC card if possible) What made you decide to start at that time in the pregnancy?
- Some people say ANC is not necessary if a woman is healthy. Other people say every pregnant woman should get ANC. What do you think?
- Is there a time in pregnancy that’s too early to start ANC? (Why is it too early? What makes it too early? What happens if a pregnant woman goes to ANC too soon?)

1. Now I would like you to tell me about malaria here in [name of village]. How big a problem is malaria for people in [name of village]?
   - Who (what types of people) is most affected by malaria in [name of village]?
   - What does your family do to prevent malaria? *Not sure if we need this*
2. Now I’d like you tell me about pregnant women and malaria in [name of village]. How important a problem is malaria for pregnant women in [name of village]?

- Some people say that getting malaria when you are pregnant is the same as getting malaria any other time. Some people say getting malaria is more dangerous when you’re pregnant. What do you think? (Probe thoroughly to try to overcome potential social desirability bias)
- Have you ever had malaria when you’re pregnant? (Have you had malaria during this pregnancy?) What happened?
- What can a pregnant woman do to prevent getting malaria?

1. Some CSComs give pregnant women pills to prevent malaria. Does [name of local CSCom] give pills to pregnant women to prevent malaria? Has [name of local CSCom] ever offered you pills to prevent malaria? If so, what are the pills like? What was your experience with these pills?
   - What made you decide to take the pills / not take the pills?
   - When in your pregnancy did you take the pills?
   - How often / how many times have you taken the pills?
   - What can you tell me about taking the pills? If your sister or friend asked you if they should take the pills when they’re pregnant, what would you tell them?
   - What are some reasons why a pregnant woman might not want to take the pills?
2. The Ministry of Health says women should take these pills 3 or 4 times during pregnancy to prevent malaria. But many women take the pills only one time or not at all. What are the reasons why most women don’t take the pills as often as the Ministry recommends?
   - What stops women from taking the pills? Why do many women not take the pills?
   - What would make pregnant women more likely/willing to take these pills?
3. Some people sleep under mosquito nets treated with insecticide to prevent malaria. What experience have you had sleeping under an insecticide-treated mosquito net?
   - What do you like and dislike about ITNs?
   - Are there certain times when people cannot use ITNs? What are those times? Why can’t people use ITNs at those times?
   - What is it like to use an ITN when you are pregnant? What would make a pregnant woman want to use an ITN or not want to use an ITN?
   - We have noticed that some pregnant women sleep under nets and some do not. Can you help us understand why?
4. What else is important to know about malaria during pregnancy in [name of village]?

**Instrument #2: Interview/focus group guide for husbands, mother-in-laws, family members**

*Note to interviewer: Explain to your informant that you are interested in their experience with their family member’s pregnancy. Explain why they was chosen to be interviewed and that their perspective is very important to you. Emphasize that you want to learn from them, that there are no right or wrong answers, and that they can interrupt you at any time if they have something to say. Let the informant talk about what they thinks is important. Remind them that you will not record their name and that everything you talk about will be kept confidential.*

1. I’d like to start off by asking you about health during pregnancy. What are the important things for a woman to do to stay healthy when she is pregnant?

Probes:

- What should a woman do to protect her health when she is pregnant?
- What should she do to make sure her baby is born healthy?
- Is there any kind of special care a woman needs when she is pregnant?
- Where does she get that care? (If appropriate, ask about different kinds of care for different things to try to determine if there are certain health issues the informant identifies as appropriate domains for biomedical care versus others that are appropriate domains for other kinds of care.)
- In addition to the pregnant woman herself, who helps decide about care during pregnancy?
- What happens if people who help decide are away or cannot be reached?

1. What illnesses can happen during pregnancy?

Probes:

- What illnesses are especially dangerous for the mother or the baby?
- What makes them especially dangerous?
- What can/should a pregnant woman do to keep from getting [name of illness]?
- If a pregnant woman got [name of illness] what should she do?

1. Some people say it’s good to take certain medicines during pregnancy. Some people say pregnant women shouldn’t take medicine. What do you think?

*If the interviewee mentions a particular medicine, probe as appropriate: What does this medicine do? What is the purpose of taking it? How often should it be taken?*

Probes:

- Has your wife taken any medicines during her pregnancy?
- What was her experience with this medication? What, if anything, happened when she took it?

*(We are interested in concerns about adverse effects, preferably without having to prompt directly)*

- What medicines should women avoid when they are pregnant?
- Why is it important to avoid these medicines?
- What are the occasions/reasons when a woman should take medicine during pregnancy?
- Are there medicines a pregnant woman who is healthy should take to keep from getting sick? If so, when should she take medicine to keep from getting sick?
- In addition to the pregnant woman herself, who helps decide about using medication during pregnancy? What is that person is away or cannot be reached?

1. Now I’d like to ask you about the care for pregnant women provided by the Health Center (CSCom). You may know that the CSCom provides a special type of care for pregnant women called antenatal care (ANC). Some people say ANC is not necessary if a woman is healthy. Other people say every pregnant woman should get ANC. What do you think?

Probes:

- If your brother or a friend asked you if his wife should go to ANC, what would you tell him?
- What are some reasons a pregnant woman might go to ANC? What are some reasons a pregnant woman might not go to ANC?
- When should a pregnant woman make her first ANC visit? What makes that the right time?
- Is there a time in pregnancy that’s too early to start ANC? (Why is it too early? What makes it too early? What happens if a pregnant woman goes to ANC too soon?)
- Besides the CSCom, where else could a pregnant woman go for care during pregnancy?
- (As a follow-up to the previous probe): You mentioned X, Y, and Z as places a woman might go for care during pregnancy. How would a woman decide whether / when to go to X, when to go to Y, and when to go to Z?

1. Now I would like you to tell me about malaria here in [name of village]. How big a problem is malaria for people in [name of village]?

- Who is (what types of people are) most affected by malaria in [name of village]?
- What does your family do to prevent malaria? *Not sure if we need this*
- How important a problem is malaria for pregnant women in [name of village]?
- Some people say that getting malaria when a woman is pregnant is the same as getting malaria any other time. Some people say getting malaria is more dangerous for a pregnant woman. What do you think? (Probe thoroughly to try to overcome potential social desirability bias)
- What can a pregnant woman do to prevent getting malaria?

1. Some CSComs give pregnant women pills to prevent malaria. Does [name of local CSCom] give pills to pregnant women to prevent malaria? Has [name of local CSCom] ever offered your wife pills to prevent malaria? What do you think about these pills?
   - Should pregnant women take the pills? Why?
   - If your brother or a friend asked you if his wife should take the pills, what would you tell them?
   - If a woman is going to take the pills, are there particular times in her pregnancy when she should or shouldn’t take them?
   - How often / how many times should she take them?
   - What are some reasons why a pregnant woman might not [want to] take the pills?
2. The Ministry of Health says women should take these pills 3 or 4 times during pregnancy to prevent malaria. But many women take the pills only one time or not at all. What are some reasons why women don’t take the pills as often as the Ministry recommends?
   - What stops women from taking the pills? Why do many women not take the pills?
   - What would make pregnant women more likely/willing to take these pills?
3. In some places, the CSCom provides mosquito nets treated with insecticide to pregnant women during antenatal care. Does the CSCom in [village] provide nets to pregnant women?
   1. Are they free or do women have to pay something?
   2. Are they available all the time or only some times? How often are they available?
   3. Should pregnant women sleep under an ITN? Why or why not?
   4. We have noticed that some pregnant women sleep under nets and some do not. Can you help us understand why?
4. What else is important to know about malaria during pregnancy in [name of village]?

**Instrument #3: Interview/focus group guide for village leaders**

*Note to interviewer: Explain your informants that you are interested in learning about pregnancy in this village. Explain why they were chosen to be interviewed and that their perspective is very important to you. Emphasize that you want to learn from them, that there are no right or wrong answers, and that they can interrupt you at any time if they has something to say. Let informants talk about what they think is important. Remind them that you will not record their names and that everything you talk about will be kept confidential.*

1. Please tell me a little bit about yourself and how you came to live/work in this [name of village]. How long have you been a teacher/headman/[name of position]? What brought you to [name of village]? How long have you lived here?
2. We are interested in understanding more about pregnancy in [name of village]. In particular, we are interested in knowing what women and families do to ensure the well-being of the pregnant woman and the child she is carrying. Since you are an educated person/leader/other in [name of village], I am hoping you can help me understand the different things women, husbands/fathers, and other family members do when a woman is pregnant. In most places, women and their husbands and other family members do different things to help a woman stay well during her pregnancy and give birth to a healthy child. They do different things to protect the woman and her baby from harm. Please tell me about what people do here: what kinds of harm or misfortune would a pregnant woman and her family be concerned about? What would a pregnant woman or other members of her family do to protect herself and her baby against harm?
   - What are some specific things women do to stay well during pregnancy? What are some specific things women do protect themselves and their babies against harm or misfortune?
   - What are some specific things women avoid doing to stay well during pregnancy? What are some specific things women avoid doing to protect themselves and their babies against harm?
   - When would a woman tell her husband that she was pregnant? When would a woman tell other family members? When would she let neighbors, friends and other community members know?
   - Who cares for pregnant women who live in [village]? What sort of care do they provide? Who else provides care? What sort of care do they provide? Etc.
   - If a pregnant woman needs to ward off misfortune in order to protect her pregnancy, who could she go to for help?
   - You mentioned X, Y, and Z as places a woman might go for care during pregnancy. How would a woman decide whether / when to go to X, when to go to Y, and when to go to Z? Under what circumstances would she go to X? Under what circumstances would she go to Y? Under what circumstances would she go to Z?
   - What are some reasons a woman might hesitate/might be reluctant to seek care from X/Y/Z?
3. What illnesses do pregnant women have in [village]?

Probes:

- What illnesses do people in [village] consider especially dangerous for the mother or the baby?
- What makes them especially dangerous?
- What kinds of things do pregnant woman in [village] do to keep from getting [name of illness]?
- If a pregnant woman in [village] got [illness] what would she do?

1. Some people say it’s good to take certain medicines during pregnancy. Some people say pregnant women shouldn’t take medicine. What do people in [village] say about this?

*If the interviewee mentions a particular medicine, probe as appropriate: What does this medicine do? What is the purpose of taking it? How often should it be taken?*

- What medicines do women in [village] avoid when they are pregnant?
- Why do they avoid these medicines?
- What are the occasions/reasons when a pregnant woman in [village] would take medicine?
- Are there medicines that a *healthy* pregnant woman in [village] might take to keep from getting sick? If so, when would she take that medicine?
- In addition to the pregnant woman herself, who helps decide about using medication during pregnancy? What if that person is away or cannot be reached?

1. Now I would like to ask you about malaria here in [name of village]. How big a problem is malaria for people in [name of village]?

- How important a problem is malaria for pregnant women in [name of village]?
- Some people say that getting malaria when a woman is pregnant is the same as getting malaria any other time. Some people say getting malaria is more dangerous for a pregnant woman. What do you think? (Probe thoroughly to try to overcome potential social desirability bias)
- What can do pregnant woman in [village] do to avoid getting malaria?

1. Some CSComs give pregnant women pills to prevent malaria. Does [name of local CSCom] give pills to pregnant women to prevent malaria? Has anyone in the village ever said anything to you about pills to prevent malaria during pregnancy? What did they say?
   - Do people in [village] think that pregnant women should take the pills? Why?
   - If a woman is going to take the pills, are there particular times in her pregnancy when she should or should not take them?
   - How often / how many times should she take them?
   - What are some reasons why a pregnant woman might not [want to] take the pills?
2. The Ministry of Health says women should take these pills 3 or 4 times during pregnancy to prevent malaria. But many women take the pills only one time or not at all. What are some reasons why women don’t take the pills as often as the Ministry recommends?
   - What stops women from taking the pills? Why do many women not take the pills?
   - What would make pregnant women more likely/willing to take these pills?
3. In some places, the CSCom provides mosquito nets treated with insecticide to pregnant women during antenatal care. Does the CSCom in [village] provide nets to pregnant women?
   - Are they free or do women have to pay something?
   - Are they available all the time or only some times? How often are they available?
   - Should pregnant women sleep under an ITN? Why or why not?
   - We have noticed that some pregnant women sleep under nets and some do not. Can you help us understand why?
4. What else is important to know about malaria during pregnancy in [name of village]?

**Instrument #4: Interview guide for village-level health workers**

We are doing a study on care for pregnant women in Mali, including in [name of health zone]. We are want to learn more about antenatal care and about malaria in pregnancy.

1. First, I’d like to ask you about ANC here in [name of health center]. Could you please tell me about what happens when a pregnant woman comes for ANC: what happens on the first visit – what exams and tests she receives, what counseling she receives, what medicine she receives, and so on – everything that happens on a first visit. Could you walk me through what happens from the time the woman arrives for her first visit until she leaves? If I were a pregnant woman arriving for my first visit, where would I go, who would see me first, what would I do, who would see me next, and so on – from the time I arrived until the visit was over.

*Note: probe based on specifics in the initial question*

1. Now could you tell me what happens on the second visit? If I were a pregnant woman coming for my second visit, where would I go, what would I do, who would see me first, who would see me next, what exams and tests I would have, and so on – just like you told me about the first visit.

*Note: probe as in question 1. Then ask about subsequent visits.*

1. Now I’d like to talk about malaria in pregnant women. How big a problem is malaria in pregnancy here in [name of health zone]?

*Use the following probes if they have not come up previously*

- 1. How does ANC address malaria in pregnancy? What is done during ANC to prevent malaria in pregnancy? If I were a pregnant woman coming for ANC, what would you tell me about malaria in pregnancy? What would you want me to do?
  2. Is malaria prevention a part of ANC? If so, what malaria prevention measures are included in ANC?

*Adjust the wording of the following questions as appropriate depending on answers to the previous questions.*

1. Some people recommend giving medicine to pregnant women to prevent them from getting malaria. Other people say that you only need medicine if you are sick. What do you think? Should pregnant women receive medicine to prevent malaria even if they don’t have malaria?
   1. Is it safe to give pregnant women medicine to prevent malaria? (If not, what are the dangers?)
   2. If you give pregnant women medicine to prevent malaria here, what medicine do you give?
   3. How often is [name of medicine] available? How often is it not available/out of stock? When you run out, what do you do to get more?

*Note: probe as much as possible to learn as much detail as possible about supply chain: Do they reorder before they run out or only after? Is there a regular resupply system or do they have to request it? Who do they contact to request it? How do they contact that person? (e.g., by phone, by visit, by written procurement request, etc.?) Once they request, how long does it take before they receive a shipment? Can they request resupply any time or only at certain times (e.g., the beginning of the month)? Etc., etc.*

- 1. At what point in the pregnancy do you give the medicine? Is there any time when you should not give it? If so, when? Why should you not give it at those times?
  2. Are there other reasons not to give pregnant women medicine to prevent malaria? If so, what are they?
  3. Do women have to pay anything for the medicine?
  4. How often (how many) times do you give the medicine?
  5. How do you keep track of how many times a woman has received the medicine? Is it possible for a woman to receive the medicine more than once without the health record showing it?

*Continue if appropriate*

1. What do you see as the most important reasons why some pregnant women do not receive any IPTp – not even one dose?
   1. What are the most important barriers related to the health system?
   2. What could the health system do to increase the percentage of pregnant women receiving at least one dose of IPTp?
   3. What are the most important barriers related to the community and the family?
   4. What should be done at the community and the family level to increase the percentage of women receiving at least one dose of IPTp?
2. What do you see as the most important reasons why pregnant women who receive their first dose of IPTp do not receive a second or third dose?
   1. What are the most important barriers related to the health system?
   2. What could the health system do to increase the percentage of pregnant women receiving two or more doses of IPTp?
   3. What are the most important barriers related to the community and the family?
   4. What should be done at the community and the family level to increase the percentage of women receiving two or more doses of IPTp?
3. What do you see as the principal barriers to consistent LLIN use during pregnancy?

- How do issues in the availability of LLINs impact consistent use during pregnancy? How often do issues like this occur? Are they predictable?
- Do you feel that women in your [village, region, nation] express a willingness to use LLINs? What makes you say this? Is there a general consensus on use of this medicine, or is there dispute?

1. What else is important to know about malaria during pregnancy [in name of village, district, region]?

**Instrument #5: Interview guide for Regional and District level health officials**

As you probably know, the government of Mali recommends at least 2 doses of intermittent preventive treatment for malaria in pregnancy for all pregnant women. As you probably also know, it has been difficult to achieve that goal. We’re doing this study to try to better understand how IPTp works in Mali. In addition to understanding how women are supposed to receive IPTp, we are trying to understand the barriers to increasing IPTp coverage. By understanding how IPTp is delivered and the barriers to IPTp, our goal is to figure out how to improve IPTp coverage and help Mali achieve its goal of 2 doses for 100% of pregnant women.

1. First, could you please help me understand how pregnant women receive IPTp in the current health system? I know that IPTp is provided through the local health facility – the CSCom or CSRef – but can you help me understand everything that has to happen before the woman arrives at the facility?

*Note: Give the informant some time to respond to this first question before using specific probes below. Also, probe on the respondent’s spontaneous answers before moving on the specific probes.*

- 1. Who is responsible for implementing the policy? (What units in the Ministry of Health? How well do they coordinate with one another? What are the challenges to coordinating with one another?)
     1. What is the role of the PNLP?
     2. What is the role of the department of maternal and child health?
  2. Who administers the medicine to the woman at the health facility (what class of health worker?) How are they trained? Who is responsible for training? What percentage of [cadre] has been trained nationwide/in this region/in this district? How are they supervised? Who is responsible for supervising?
  3. How are records about IPTp kept for a pregnant woman? How do you know how many doses she has received? How does this information get from the health facility to the district, regional, and national level? What are the strengths and weaknesses of the record-keeping system (HMIS)?
  4. Who purchases the SP, how does the SP get from central medical stores to the region and the district and the health facility? How does a CSCom obtain additional supplies of SP when its stock runs low?

Now that we’ve discussed how IPTp is delivered, I’d like to ask you about barriers to pregnant women receiving IPTp.

1. What do you see as the most important reasons why some pregnant women do not receive any IPTp – not even one dose?
   1. What are the most important barriers related to the health system?
   2. What could the health system do to increase the percentage of pregnant women receiving at least one dose of IPTp?
   3. What are the most important barriers related to the community and the family?
   4. What should be done at the community and the family level to increase the percentage of women receiving at least one dose of IPTp?
2. What do you see as the most important reasons why pregnant women who receive their first dose of IPTp do not receive a second or third dose?
   1. What are the most important barriers related to the health system?
   2. What could the health system do to increase the percentage of pregnant women receiving two or more doses of IPTp?
   3. What are the most important barriers related to the community and the family?
   4. What should be done at the community and the family level to increase the percentage of women receiving two or more doses of IPTp?
3. As a health official, what do you see as the principal barriers to consistent LLIN use during pregnancy?

- How do issues in the availability of LLINs impact consistent use during pregnancy? How often do issues like this occur? Are they predictable?
- Do you feel that women in your [village, region, nation] express a willingness to use LLINs? What makes you say this? Is there a general consensus on use of this medicine, or is there dispute?

1. What else is important to know about malaria during pregnancy [in name of village, district, region]?
